# Neonatal Fc receptor is involved in the protection of fibrinogen after its intake in peripheral blood mononuclear cells

**DOI:** 10.1186/s12967-018-1446-2

**Published:** 2018-03-14

**Authors:** Tiziana Alberio, Greta Forlani, Marta Lualdi, Giovanna Tosi, Roberto S. Accolla, Mauro Fasano

**Affiliations:** 1 Department of Science and High Technology, University of Insubria, Via Manara, 7, 21052 Busto Arsizio, VA Italy; 2 Center of Neuroscience, University of Insubria, Busto Arsizio, Italy; 3 Center of Bioinformatics, University of Insubria, Como, Italy; 4 Department of Medicine and Surgery, University of Insubria, Via Ottorino Rossi, 9, 21100 Varese, Italy

**Keywords:** Fibrinogen, Neonatal Fc receptor, T-cells

## Abstract

**Background:**

Fibrinogen is a central player in the blood coagulation cascade and one of the most abundant plasma proteins. This glycoprotein also triggers important events (e.g., cell spreading, the respiratory burst and degranulation) in neutrophil cells via a α_M_β_2_ integrin-mediated binding to the cell surface. Yet, little is known about the interaction of fibrinogen with leukocytes other than neutrophils or stimulated monocytes, although high amounts of fibrinogen protein can also be found in lymphocytes, particularly in T-cells. The aim of the present work is to unveil the dynamics and the function of fibrinogen intake in T-cells.

**Methods:**

Using the Jurkat cell line as a T-cells model we performed fibrinogen intake/competition experiments. Moreover, by means of a targeted gene knock-down by RNA-interference, we investigated the dynamics of the intake mechanism.

**Results:**

Here we show that (i) fibrinogen, although not expressed in human peripheral blood mononuclear cells, can be internalized by these cells; (ii) fibrinogen internalization curves show a hyperbolic behavior, which is affected by the presence of serum in the medium, (iii) FITC-conjugated fibrinogen is released and re-internalized by adjacent cells, (iv) the presence of human serum albumin (HSA) or immunoglobulin G (IgG), which are both protected from intracellular degradation by the interaction with the neonatal Fc receptor (FcRn), results in a decreased amount of internalized fibrinogen, and (v) FcRn-knockdown affects the dynamics of fibrinogen internalization.

**Conclusions:**

We demonstrated here for the first time that fibrinogen can be internalized and released by T-lymphocyte cells. Moreover, we showed that the presence of serum, HSA or IgG in the culture medium results in a reduction of the amount of internalized fibrinogen in these cells. Thus, we obtained experimental evidence for the expression of FcRn in T-lymphocyte cells and we propose this receptor as involved in the protection of fibrinogen from intracellular lysosomal degradation.

**Electronic supplementary material:**

The online version of this article (10.1186/s12967-018-1446-2) contains supplementary material, which is available to authorized users.

## Background

Fibrinogen is one of the most abundant plasma proteins, with a concentration of about 10 μM (340 mg/dl) in healthy subjects [1]. This plasma glycoprotein is synthesized mainly in hepatocytes through the secretory pathway and it is comprised of two sets of three polypeptide chains (namely Aα, Bβ and γ) of 610, 461 and 411 residues, respectively, joined together by disulfide bridges [2]. Fibrinogen plays a central role in the blood coagulation cascade, which is triggered by the conversion of fibrinogen to fibrin by limited proteolysis. Additionally, fibrinogen expression is induced by IL-6 as part of the acute phase reaction. Eventually, fibrinogen participates, together with fibrin, in several biological processes including fibrinolysis, cellular and matrix interactions, inflammation and wound healing [1–3].

In severe inflammatory conditions the plasma concentration of fibrinogen can increase up to 10 mg/ml [4] and this is necessary for processes other than coagulation. Indeed, fibrinogen binds to integrin α_M_β_2_ (Mac-1) on both neutrophil and stimulated monocyte cells surface, thus mediating enhanced binding of leukocytes at sites of damaged endothelium and promoting leukocyte extravasation [4–6]. The binding of fibrinogen to neutrophil β_2_ integrin receptors induces the tyrosine phosphorylation of neutrophil proteins which, in turn, provides the signal for the initiation of various important cellular events, such as cell spreading, the respiratory burst and degranulation [7]. Eventually, it has been suggested that the interaction of fibrinogen with neutrophils may also lead to its degradation after internalization by non-specific pinocytosis [7].

Interestingly, fibrinogen protein β and γ chains have been also found in isolated lymphocytes [8], particularly in T-cells [9], in which the observed amounts were relevant when compared to the most abundant cytoskeletal protein β-actin. Nevertheless, to date nothing is known about the interaction of fibrinogen with leukocytes other than neutrophils and monocytes.

The neonatal Fc receptor (FcRn) has been initially identified as the receptor involved in the IgG transmission from mother to offspring [10, 11]. Subsequently, it has been shown that FcRn is expressed in many tissues and cell types beyond neonatal life [12], including polarized epithelia (intestines, lung, breast, kidney) as well as parenchymal cells (hepatocytes, endothelial cells and hematopoietic cells) [13, 14]. FcRn receptor has been extensively characterized, thus unveiling important roles in several biological functions. Indeed, FcRn transports IgG across epithelia [15–17], provides passive immunity to the newborn and also participates in the development of the adaptive immune system [18, 19] and is deeply involved in the intracellular trafficking of IgG through the endolysosomal pathway [20, 21]. In particular, FcRn binds IgG with high affinity at low pH, a way to prolong IgG half-life by preventing in part their lysosomal degradation [22–24].

It is interesting that FcRn was recognized as the receptor of albumin, another plasma protein with long half-life [23, 25]. In this case albumin is internalized by pinocytosis and subsequently bound by FcRn in the acidic pH environment of the early endosome, thus rescuing it from degradation when the complex migrates to the lysosome [26]. Albumin is then released by exocytosis in the extracellular space, where the neutral pH counteracts its binding to the FcRn receptor [27].

Here, we report on the dynamics of internalization of fibrinogen within lymphocyte cells and demonstrate that FcRn is involved in the rescue of fibrinogen from lysosomal degradation.

## Methods

### Non-T and T-cells fractions preparation from peripheral blood

Five volunteers have been enrolled by the Department of Neuroscience, University of Torino and signed an informed consent before being recruited, following approval by the Institutional Review Board of the University Hospital and according to the *Declaration of Helsinki*. The subjects (3 females and 2 males; average age: 54 ± 9 years) underwent a venous blood sampling (20 ml) from the antecubital vein, between 9 and 10 a.m., after an overnight fast. The five subjects declared no inflammatory diseases and/or drug treatments within 2 weeks before sampling. Whole blood was collected into vacuum tubes containing EDTA, diluted with 50 ml of phosphate-buffered saline (PBS) and stratified in two 50 ml tubes on top of 15 ml of Lympholyte^®^-H (Cedarlane, Burlington, Canada) each. After centrifugation (800×*g*, 20 min, 20 °C), peripheral blood mononuclear cells (PBMCs) were collected, centrifuged at 400×*g*, 15 min, 20 °C and washed twice with 10 ml of magnetic-activated cell sorting (MACS) buffer (Miltenyi Biotec, Cologne, Germany). The isolation of T-cells was achieved by MACS with the Pan T cell isolation kit (Miltenyi Biotec) using the manufacturer’s protocol. The non-T fractions (all PBMC but T-cells) have been also collected.

### Cells and treatments

The Jurkat T-cell leukemia cells (kindly provided by Prof. Jean-Pierre Mach, University of Lausanne, Switzerland) were maintained at 37 °C in a 5% CO_2_ humidified atmosphere in RPMI 1640, supplemented with 10% fetal bovine serum (FBS), 100 U/ml penicillin, 100 μg/ml streptomycin, and 2 mM l-glutamine. Human neuroblastoma SH-SY5Y (ECACC 94030304) cell line was maintained at 37 °C in a 5% CO_2_ humidified atmosphere in DMEM, supplemented with 10% FBS, 100 U/ml penicillin, 100 μg/ml streptomycin, and 2 mM l-glutamine. All cell culture media and reagents were from Euroclone (Pero, Milano, Italy). Stock flasks were transferred for culture twice weekly or as required to maintain optimal cell growth.

For the equilibrium intake experiments, Jurkat cells were incubated with 3, 2, 1, 0.6, 0.3, 0.2, 0.15, 0.1, 0.05 mg/ml of fibrinogen (Axxora, NZY-F004-M050) in RPMI 1640, with or w/o FBS, for 4 h.

For the intake kinetics experiments, Jurkat cells were incubated with 0.4 mg/ml fibrinogen for 0.5, 1, 4, 6, 24 h in RPMI 1640, with or w/o FBS.

For the concomitant incubation with fibrinogen and human serum albumin (HSA), IgG, hemoglobin or catalase, Jurkat cells have been incubated for 4 h with 0.4 mg/ml fibrinogen and 0.4 mg/ml HSA, 50 µg/10^6^ cells of IgG, hemoglobin or catalase, respectively.

Enzymatic protein deglycosylation was performed using the PNGase F enzyme, following the manufacturers’ instructions (EDEGLY kit, Sigma-Aldrich). Briefly, total cell lysates from SH-SY5Y cells were clarified by protein precipitation (acetone/methanol) and proteins were resuspended in 200 mM HEPES/NaOH. After protein quantification, 100 µg of total proteins were used for the deglycosylation reaction (24 h, 37 °C).

### Western blotting analysis

Cells were collected by centrifugation (300*g*, 5 min, 25 °C) and resuspended in RIPA buffer (25 mM Tris–HCl, pH 7.4, 150 mM NaCl, 1% Triton X-100, 1% SDS, 1% sodium deoxycholate) in the presence of protease inhibitors (Sigma-Aldrich) for 30 min, 4 °C, under shaking. Extracts were cleared by centrifugation (10,000*g*, 30 min, 4 °C) and protein concentration in the supernatants was determined spectrophotometrically with the bicinchoninic acid assay (Thermo Fisher, Waltham, Massachusetts). Cell lysates (20 µg) were denatured in Laemmli sample buffer for 5 min at 98 °C and electrophoresed on 10% SDS-PAGE gel. Proteins were transferred to polyvinylidenedifluoride (PVDF) membranes at 1 mA/cm^2^, 1.5 h (TE77pwr, Hoefer, Holliston, MA). Membranes were saturated in 5% non-fat milk in TBS-T (0.1 M Tris–HCl pH 7.4, 1.5 M NaCl and 0.5% Tween-20) and incubated in the same buffer at 4 °C overnight with primary antibodies: goat anti-fibrinogen polyclonal antibody (PAB11318, Abnova, Taiwan), 1:10,000 dilution; mouse anti-beta actin monoclonal antibody (GTX23280, GeneTex, San Antonio, Texas), 1:8000 dilution; rabbit anti-FCGRT polyclonal antibody (Sigma-Aldrich, HPA012122), 1:500 dilution; rabbit anti-HSA polyclonal antibody (Sigma-Aldrich, HPA031025), 1:10,000 dilution. Membranes were then washed with TBS-T and incubated in 5% milk-TBS-T with peroxidase-conjugated secondary antibodies: rabbit anti-goat IgG antibody (AP106P, Millipore, Billerica, Massachusetts), 1:10,000 dilution; Pierce^®^ goat anti-mouse IgG antibody (Thermo Scientific, 31432), 1:8000 dilution; goat anti-rabbit IgG antibody (Millipore, AP132P) 1:2000–1:10,000 dilution. Chemiluminescence detection was performed following manufacturer’s instructions (Millipore, WBKLS0500). Images (16 bit grayscale) were acquired with the G:BOXChemi XT4 (Syngene, Cambridge, UK) system and analyzed using the ImageJ software (http://rsb.info.nih.gov/ij/). Signal intensities were corrected for protein loading by normalization to β-actin intensity. Statistical significance was verified by Student’s *t*-test with Welch correction for heteroscedasticity.

### Co-culture and flow cytofluorimetric analysis (FACS)

Jurkat or SH-SY5Y cells were loaded with Fibrinogen-FITC (Axxora, NZY-F006) for 4 h at 37 °C (0.4 mg/ml in RPMI medium w/o FBS). Loaded cells were washed three times with RPMI w/o FBS and the fibrinogen uptake was analyzed by flow cytometry (EPICS XL flow cytometer) and EXPO32 analysis software (Beckman Coulter, Pasadena, California) at different time points as previously described [28].

For co-culture experiments, 20% of untreated Jurkat cells were mixed with 80% of Fibrinogen-FITC-loaded Jurkat cells in RPMI medium w/o FBS. Cells were analyzed by flow cytometry as indicated above.

### RNA-interference assay

SH-SY5Y cells were seeded in 12-well plate and transfected with Lipofectamine^®^ MessengerMAX™ reagent (Life Technologies, Carlsbad, California) using 100 nM siRNAs (final concentration) according to the manufacturer’s instructions.

The following siRNAs from Dharmacon (Lafayette, Colorado) were used: ON-TARGETplus human FCGRT (2217) siRNA SMARTpool (siRNA J-017906-05 targeting CGUCAUCGGUGUCUUGCUA sequence; siRNA J-017906-06 targeting GGCGAGGAGUUCAUGAAUU sequence; siRNA J-017906-07 targeting GCGAUGAGCACCACUACUG sequence; siRNA J-017906-08 targeting GGAGCUCUGUUGUGGAGAA sequence), and ON-TARGETplus non-targeting pool D-001810-10-05 (target sequences: UGGUUUACAUGUCGACUAA, UGGUUUACAUGUUGUGUGA, UGGUUUACAUGUUUUCUGA, UGGUUUACAUGUUUUCCUA) as control.

When incubation with either HSA or fibrinogen was performed, 44 h after transfection the cells were starved in fresh serum-free medium for 1 h and then incubated with fibrinogen or HSA (0.4 mg/ml in DMEM w/o FBS) for 3 h. After incubation (48 h after transfection), the cells were washed in ice-cold PBS and harvested for western blotting and qRT-PCR assays. Three independent knock-down experiments were performed.

### RNA extraction, reverse transcription and PCR

Total RNA was extracted from SH-SY5Y, T-cells and PBMCs of the same control subject and from Jurkat and HepG2 cells using the ReliaPrep™ RNA cell miniprep system (Z6011, Promega, Milano, Italy) following the manufacturer’s instructions. Two micrograms of DNA-free total RNA were reverse transcribed into first-strand cDNA with random primers in a 20 μl final volume using the GoScript™ reverse transcription system (Promega, A5000).

Primer design was performed by using Primer3 software (http://primer3.sourceforge.net/) and manually adjusted, if needed, to avoid the amplification of undesired sequences and to have comparable melting temperature and reaction efficiency. Primer specificity was tested by BLAST (http://blast.ncbi.nlm.nih.gov/Blast.cgi) and experimentally by the positive control amplification. Primer pairs sequences: *ACTB Fw*: 5′-CAGCCATGTACGTTGCTATCCAGG-3′, *ACTB Rev*: 5′-AGGTCCAGACGCAGGATGGCATGG-3′, amplicon size: 151 bp; *FGB Fw*: 5′-TGGCAAAAGAGGCAGAAGCAAG-3′, *FGB Rev*: 5′-CCAGGATTGAACGAAGCACACG-3′, amplicon size: 145 bp; *FCGRT Fw*: 5′-ACTTCGGTTCCTGCGGAAT-3′, *FCGRT Rev*: 5′-CTGCCGTGAGTAGCAAGACA-3′, amplicon size: 242 bp; *GAPDH Fw*: 5′-GAGTCAACGGATTTGGTCGT-3′, *GAPDH Rev*: 5′-TTGATTTTGGAGGGATCTCG-3′, amplicon size: 238 bp.

The semiquantitative RT-PCR was performed using 20 ng of cDNA as template and the amplification products were separated on a 2% agarose gel stained with ethidium bromide and acquired.

The relative gene-expression qPCR analysis was performed in triplicate using 20 ng of cDNA in 25 μl final volume/well in 96-well plates, using the GoTaq^®^ qPCR master mix (Promega, A6001) following the manufacturer’s instructions. The ABI PRISM 7000 sequence detection system (Thermo Fisher) was used as real-time PCR instrument. Beta-actin (ACTB) was quantified as reference housekeeping gene. The amplification steps were set as follows: a first step at 95 °C for 10 min, 40 cycles (95 °C for 15 s, 60 °C for 1 min) and a final dissociation step (95 °C for 15 s, 60 °C for 20 s, 95 °C for 15 s). The relative expression levels of the FcRn transcript were calculated using the ΔΔCt method. Statistical significance was verified by Student’s *t*-test.

### Data analysis

Time-dependent cellular intake of fibrinogen was analyzed using simple exponential association: $Y = Y_{max} \,\left( {1 - e^{{ - k_{in} t}} } \right)$; where *Y*_*max*_ is the maximum fibrinogen signal observed in the experiment, *k*_*in*_ is the first-order kinetics constant for fibrinogen intake. The cell-bound fibrinogen fraction has been described by a simple equilibrium isotherm: $Y\, = \,\frac{{Y_{max} \,\left[ {fib} \right]}}{{K_{d} \, + \,\left[ {fib} \right]}}$; where *K*_d_ is the fibrinogen concentration needed to reach half-saturation and [*Fib*] is the fibrinogen concentration.

## Results

### Fibrinogen is present in PBMCs but is not synthesized by these cells

In order to assess the presence of fibrinogen in peripheral blood mononuclear cells (PBMCs), these cells have been isolated from peripheral blood of donors and then fractionated into two major components (defined here as T and non-T cells).

**Fig. 1:**
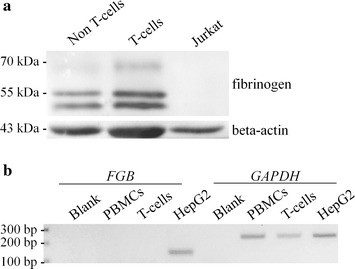
Fibrinogen expression in PBMCs. **a** A representative western blot analysis of a total cell lysate of non-T and T-cell fraction of PBMCs from the same individual (male, aged 51) and of Jurkat cell line. **b** Fibrinogen β-chain (*FGB*) mRNA expression in PBMCs and T-cells (from the same individual) and in hepatocarcinoma HepG2 cells (as positive control). After total RNA extraction, reverse transcription and semi-quantitative PCR, the amplification products (*FGB* and *GAPDH* as housekeeping control) were separated on a 2% agarose gel stained with ethidium bromide

A western blot analysis (Fig. 1a) has been performed on total cell lysates and fibrinogen resulted to be present in both the T- and the non-T cell fractions, the latter being mainly composed by B-cells, natural killer (NK) cells and monocytes. On the contrary, fibrinogen protein was not present in the human T-cell leukemia Jurkat cell line.

Thus, we verified whether the Fibrinogen β-chain (*FGB*) transcript was expressed in PBMCs by performing a semi-quantitative RT-PCR, using the human hepatocellular carcinoma HepG2 cell line as a positive control. As shown in Fig. 1b, fibrinogen β chain was not expressed in PBMCs, thus suggesting the exogenous derivation of the protein.

### Fibrinogen intake in Jurkat cells shows a hyperbolic behavior and is affected by the presence of serum in the culture medium

Since fibrinogen protein was abundantly present in PBMCs, but not expressed by these cells, we decided to assess whether the presence of fibrinogen was due to its uptake from the extracellular milieu (i.e., plasma). To this purpose, we used the Jurkat cell line, in which fibrinogen is not expressed, and we cultured these cells in medium supplemented with fibrinogen.

**Fig. 2:**
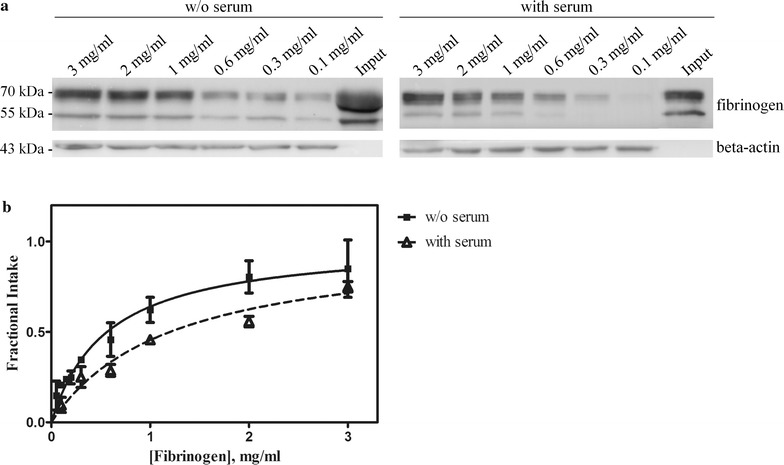
Fibrinogen intake equilibrium in Jurkat cells. **a** A western blot analysis was performed in Jurkat cells after 4 h incubation with increasing concentrations of fibrinogen in either complete or serum-free medium. Representative blots are shown. **b** The results of three independent experiments have been used to calculate the curve fit and the apparent *K*_d_ (details in the text). Error bars represents SE

Firstly, we investigated the thermodynamics and kinetics aspects of the possible intake. Jurkat cells were incubated with increasing doses of fibrinogen for 4 h, to determine the intake equilibrium. The experiments were performed either in the presence or in the absence of serum in the culture medium and, as shown in Fig. 2a, fibrinogen was incorporated into Jurkat cells and the intake showed a hyperbolic behavior, consistent with a simple equilibrium. Intake curves were generated (Fig. 2b) and the calculated apparent K_d_ in the presence of serum was 1.2 ± 0.1 mg/ml, whereas in the absence of serum an apparent K_d_ of 0.60 ± 0.15 mg/ml was observed.

**Fig. 3:**
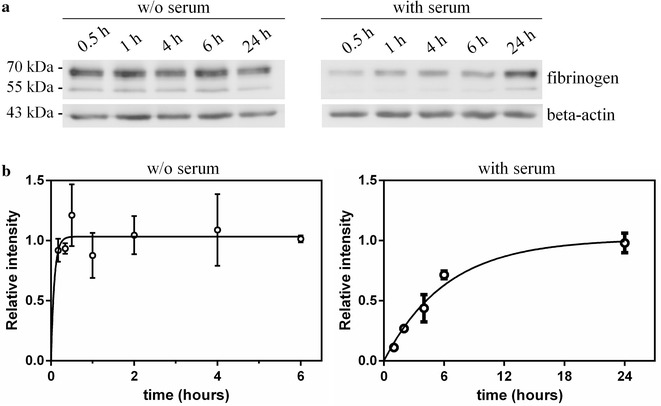
Fibrinogen intake kinetics in Jurkat cells. **a** After incubation with 0.4 mg/ml fibrinogen, total protein lysates have been obtained from Jurkat cells at different time points. Representative blots are shown. **b** The results of three independent experiments have been used to calculate the curve fit and the *k*_in_. Error bars represents SE

To assess the intake kinetics, Jurkat cells were then incubated at the same concentration of fibrinogen (0.4 mg/ml) for different time points, either in the presence or in the absence of serum. The amount of internalized protein was quantified by immunoblotting (Fig. 3a). As a result, fibrinogen intake in the absence of serum followed a fast kinetics (*k*_in_ = 12 ± 6/h) while, in the presence of serum in the culture medium, the intake showed a slower kinetics, with *k*_in_ = 0.16 ± 0.02/h (Fig. 3b).

Thus, fibrinogen can be internalized by Jurkat cells and the internalization curves show a hyperbolic behavior, which is influenced by the presence of serum in the culture medium.

### Fibrinogen is released and re-internalized by Jurkat cells

To assess the fibrinogen fate after internalization, Jurkat cells were incubated with 0.4 mg/ml fibrinogen for 4 h, washed and analyzed after a 24 h recovery period. We observed that fibrinogen was almost completely undetectable after 24 h, thus suggesting that the internalized fibrinogen was either degraded within the cells or protected from degradation and eventually released in the extracellular space.

To verify these hypotheses, we assessed the ability of Jurkat cells to secrete the internalized fibrinogen, and, if this was the case, whether it could be re-uptaken by adjacent cells. To this end, untreated Jurkat cells (R) were co-cultured with Jurkat cells pre-loaded with fluorescein isothiocyanate (FITC)-conjugated fibrinogen (S) at 1:5 ratio and analyzed by flow cytometry immediately after mixing (T0) or after 2 h (T2h) and 19 h (T19h).

**Fig. 4:**
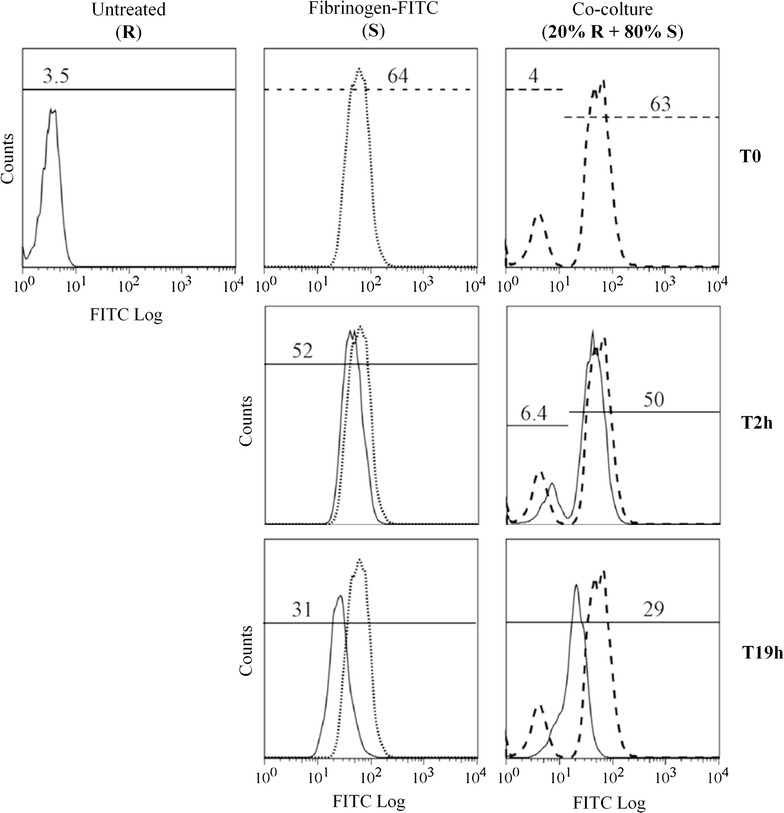
Fibrinogen-FITC exchange in Jurkat cells. Untreated Jurkat cells (R) were co-cultured in vitro with Fibrinogen-FITC-loaded Jurkat cells (S) in serum-free medium. Cells (both alone and co-cultured) were analyzed by flow cytometry immediately after mixing (T0) and then after 2 and 19 h (T2h and T19h). Histograms represent fluorescence profiles of the cells and the numbers inside the panels indicate the mean fluorescence values (m.f.) expressed as arbitrary units (a.u.)

At time zero (Fig. 4, upper panel), the fluorescence profile of the co-culture was represented by two different cell populations, one FITC-positive (corresponding to the stimulator “S” cells, about 80% of the cells) and one FITC-negative (corresponding to the responder “R” cells, about 20% of the cells). The mean fluorescence (m.f.) values of the two populations in the co-culture corresponded to that measured in the untreated cells and in the fibrinogen-FITC loaded cells cultured alone.

After 2 h (Fig. 4, middle panel) we could appreciate only a slight decrease in the amount of fibrinogen-FITC in the population of stimulator cells, as indicated by the m.f. values at T0 and T2h (64 and 52 arbitrary unit (a.u.), respectively). This reduction was accompanied by a slight shift of the fluorescence profile of the co-culture, with an increase of the m.f. value of the responder cells (from 4 to 6.4 a.u.) and a parallel decrease of the m.f. value of stimulator cells (from 63 to 50 a.u.), thus suggesting a possible exchange of fibrinogen between the two cell populations.

After 19 h (Fig. 4, lower panel), the amount of fibrinogen found in the stimulator cells was significantly lower when compared to that found at T2h, as indicated by the m.f. values (31 a.u. at T19h; 52 a.u. at T2h). Interestingly, in the co-culture we found a single cell population, in which the mean fluorescence was nearly half of that measured at T2h in the FITC-positive cells (29 a.u. with respect to 50 a.u.) and comparable to that found in the stimulator cells at 19 h (m.f. = 31), thus demonstrating that fibrinogen can be secreted and re-uptaken by Jurkat cells.

### Human serum albumin (HSA) and immunoglobulin G (IgG) modify fibrinogen internalization in Jurkat cells

Since (i) the internalization of fibrinogen was affected by the presence of serum in the culture medium, and (ii) the internalized fibrinogen was protected from intracellular degradation, released and re-uptaken from adjacent cells, we decided to perform some targeted experiments aimed at clarifying the dynamics of fibrinogen intake, transport and secretion in Jurkat cells.

To this end, we first evaluated the effects of the concomitant incubation of Jurkat cells with fibrinogen and either HSA or IgG. These proteins were selected because they are abundantly present in the serum and also because a well-characterized mechanism of rescue from degradation and recycling, mediated by the neonatal Fc receptor (FcRn), has been recently described for both of them.

**Fig. 5:**
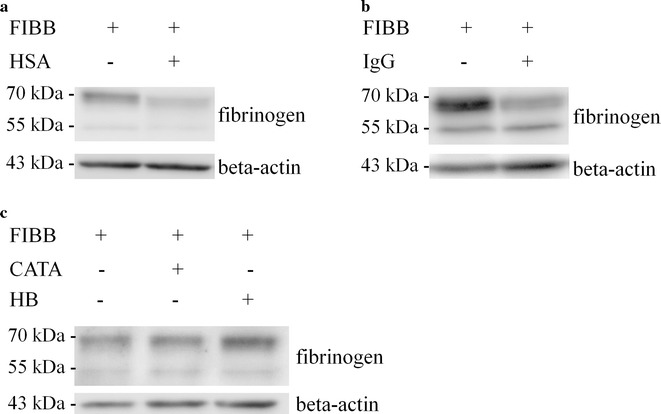
HSA and IgG influence fibrinogen internalization in Jurkat cells. A western blot analysis was performed in Jurkat cells after 4 h incubation with fibrinogen (0.4 mg/ml), in the presence or in the absence of HSA (**a**) and IgG (**b**). **c** Fibrinogen intake was also assessed in the presence of two non-plasmatic proteins, i.e., catalase (CATA) and hemoglobin (HB)

As shown in Fig. 5, the amount of internalized fibrinogen in Jurkat cells after a 4 h incubation was strongly reduced both in the presence of HSA (Fig. 5a) and IgG (Fig. 5b). Strikingly, this effect was restricted to HSA and IgG, since other non-plasmatic proteins (i.e., catalase and hemoglobin) did not affect the amount of internalized fibrinogen in Jurkat cells (Fig. 5c).

### The neonatal Fc receptor (FcRn) is involved in fibrinogen protection in SH-SY5Y cells

Since it has been demonstrated that FcRn is the receptor of both albumin and IgG, shown above to decrease the amount of internalized fibrinogen in Jurkat cells, we hypothesized that FcRn could be responsible for the protection of fibrinogen from lysosomal degradation as well. To verify this hypothesis, we first assessed the expression of FcRn in different human cell lines. In addition to Jurkat cell line as a T cells model, we used a B lymphocyte cell line (Raji), two promyelocytic cell lines (THP-1 and U-937) and a neuroblastoma cell line (SH-SY5Y). The latter represents a well-established cellular model in which high efficiency of transfection can be achieved by lipofection.

**Fig. 6:**
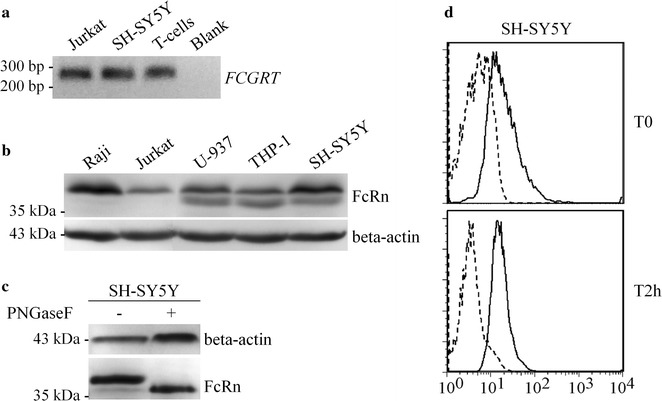
FcRn expression in diverse cell lineages. **a** RT-PCR analysis for the expression of FcRn at transcript level in Jurkat, SH-SY5Y and T-cells. **b** Western blot analysis for the expression of FcRn at protein level in Raji, Jurkat, U-937, THP-1 and SH-SY5Y cell lines. **c** Deglycosylation by PNGase F treatment on total protein lysates from SH-SY5Y cells. **d** SH-SY5Y cells were incubated with fibrinogen-FITC (0.4 mg/ml in serum-free medium) for 4 h. Cells were then washed 3 times and analyzed by flow cytometry at time zero (T0) and after 2 h (T2h). Solid histograms indicate the fibrinogen-FITC loaded cells, whereas the dashed histograms indicate the unloaded cells

FcRn resulted to be expressed in all the analyzed cell lines, as well as in T cells isolated from peripheral blood of donors (Fig. 6a, b). In particular, two distinct protein forms were detected in promyelocytic cell lines and in SH-SY5Y cells, with respect to lymphocyte cells (Fig. 6b). Since human FcRn protein can be post-translationally modified by N-glycosylation [29], we assessed whether the difference in molecular mass between the two protein forms was due to the presence of *N*-glycan moieties. To this end, we performed PNGase F treatment in SH-SY5Y total protein lysates and we observed that a single lower weight protein form was detectable after treatment, thus demonstrating that the higher molecule weight form corresponds to the *N*-glycosylated FcRn protein (Fig. 6c).

SH-SY5Y cells showed FcRn protein levels higher than Jurkat cells (Fig. 6b) and fibrinogen was neither expressed at the transcript level nor detectable at the protein level in this cell line. Moreover, SH-SY5Y cells, such as Jurkat cells, were shown to be able to uptake and release fibrinogen-FITC (Fig. 6d). Therefore, this cell line was selected for subsequent experiments.

**Fig. 7:**
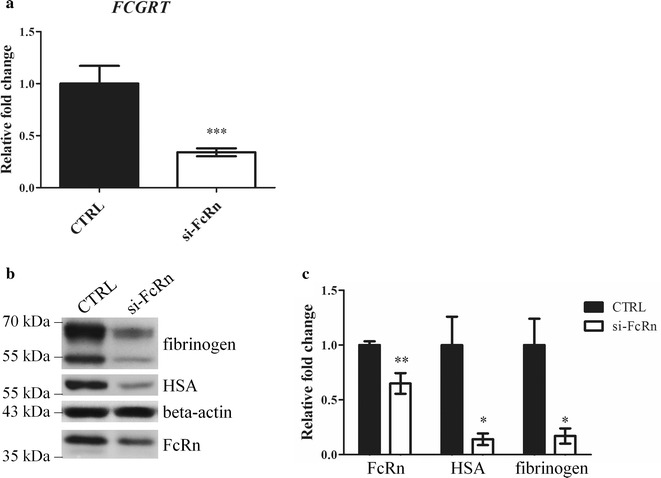
FcRn knock-down in SH-SY5Y cells. SH-SY5Y cells were transfected with either a FcRn-targeting pool of siRNAs (si-FcRn) or a control pool (CRTL) for 44 h and then incubated with 0.4 mg/ml of HSA or fibrinogen for 4 h. Three independent experiments were performed. At the transcript level (**a**) a 34% residual expression was obtained after a 48 h transfection (***p = 0.0002). At the protein level (**b**, **c**), FcRn-silenced cells showed a decrease in the FcRn protein content (**p = 0.004) accompanied by reduction of internalized HSA (*p = 0.047) and fibrinogen (*p = 0.016), when compared to control-transfected cells. **b** A representative western blot analysis is shown. **c** The results of the triplicate analysis are shown. Error bars represents SE

If the FcRn is a receptor protecting fibrinogen from degradation, the absence of FcRn is expected to cause a decrease in the amount of intracellular fibrinogen after internalization. To address this point, we performed FcRn knock-down in SH-SY5Y cells by RNA-interference and verified by qRT-PCR that the transcript level was reduced by 70% (Fig. 7a). Upon transcript depletion, we also observed a significant reduction of FcRn protein (up to 40%), as assessed by western blotting (Fig. 7b, c). Strikingly, in cells incubated with either HSA (positive control) or fibrinogen, FcRn depletion caused a significant reduction in the intracellular amount of both proteins (Fig. 7b, c), thus supporting the notion of FcRn as a receptor for fibrinogen protection as well.

## Discussion

Our previous studies clearly indicated the presence of very high levels of fibrinogen protein inside PBMCs [8, 9]. This observation was somehow surprising since fibrinogen is a highly abundant protein in plasma, with almost no roles described in peripheral cells. Therefore, we set to investigate the biochemical basis and the possible biological implications of this experimental observation.

Here, we demonstrate that fibrinogen is not synthesized by PBMCs, rather it is internalized from plasma.

In order to better characterize fibrinogen intake levels and kinetics, we took advantage of the human T cell line Jurkat. Indeed, in this working model of the major component of human PBMCs, standardized conditions can be maintained. On the contrary, PBMCs isolated from human subjects suffer from intrinsic biological variability. Jurkat cells, as T-cells in general, do not express fibrinogen, however we demonstrated that they are able to internalize it from the extracellular milieu. The fibrinogen intake in Jurkat cells showed a hyperbolic behavior, consistent with a simple equilibrium, where the presence of FBS in the medium influenced the thermodynamics of the intake. Indeed, if cells were maintained in standard medium containing FBS, the calculated apparent K_d_ was twofold compared to that obtained in the absence of serum.

Of note, fibrinogen-exposed Jurkat cells completely eliminated the protein within 24 h. Co-culture of Jurkat cells loaded or not with FITC-conjugated fibrinogen allowed us to follow the two phases of internalization and secretion of fibrinogen in an ordered sequence. These experiments not only demonstrated that fibrinogen is secreted, but also that it can be re-uptaken by adjacent cells.

As far as the internalization phase, we showed that while a very fast kinetics was observed in the absence of serum, a slower kinetics characterized the intake of fibrinogen in the presence of FBS in the culture medium. The fact that either HSA or IgG, which are both serum proteins, could mimic the presence of FBS in conditions of serum-free medium suggested that a common mechanism could orchestrate the dynamics of internalization, transport and secretion of these proteins. This was further corroborated by the fact that other non-plasmatic proteins, such as catalase or hemoglobin, did not alter fibrinogen intake in Jurkat cells.

Importantly, the evidence that fibrinogen could be internalized and re-externalized implied a mechanism of protection from intracellular degradation. Within this frame, it has been well documented by literature [22–25] that both HSA and IgG half-lives in serum result to be extended by the pH-dependent interaction with the neonatal Fc receptor (FcRn), which protects these proteins from intracellular lysosomal degradation and recycles them to the extracellular space. We thus hypothesized that FcRn could subserve the same function also for fibrinogen.

FcRn was originally identified as the receptor in charge to regulate IgG transport from a mother to fetus, thereby the definition as “neonatal” receptor [10, 11]. Hereafter, it has been related to IgG homeostasis and transport across polarized epithelial tissues also in adults. Therefore, it has been described as the receptor able to prevent or at least minimize IgG degradation in the lysosomes, being responsible for their long half-life in the serum [22–24]. IgG are internalized aspecifically by pinocytosis; however, IgG bind to FcRn upon acidification of the endosome, thus allowing IgG to escape lysosomal degradation. After vesicle docking with the plasma membrane, the pH returns to neutral with consequent release of bound IgG to the serum. The same mechanism was described to protect albumin from degradation and consequently to increase the half-life of the protein [30].

The results presented in this investigation indeed strongly support the idea that also fibrinogen, another abundant serum protein, could be internalized and then protected by degradation through a mechanism involving FcRn. A series of relevant observations in this direction were made. First, fibrinogen can be re-externalized and uptaken by neighboring cells, as demonstrated by co-culture experiments. Second, silencing of FcRn by siRNAs clearly showed a decreased accumulation of fibrinogen in SH-SY5Y cells.

FcRn interacts with IgG and albumin through residues located in opposite surfaces, such that FcRn can simultaneously bind IgG and albumin with neither competition nor cooperation occurring [31]. As for the interaction between FcRn and IgG, upon binding at pH 6, the protonation of three histidine residues (H310, H435, H436) in the C_H__2_–C_H__3_ hinge region of IgG allows for the formation of salt bridges at the FcRn-Fc interface [32]. By contrast, FcRn binding to albumin is mostly hydrophobic in nature and is stabilized by a pH-dependent hydrogen-bonding network internal to each protein. This interaction involves two tryptophan (W53, W59) residues of FcRn and three histidine (H464, H510, H535) residues of albumin [33]. In this frame, it is quite surprising that we observed a decreased amount of internalized fibrinogen in Jurkat cells in the presence of either HSA or IgG. The biochemical and structural characterization of the binding between fibrinogen and FcRn is beyond the scope of the present work. However, in order to explain mechanistically our results, it can be hypothesized that a steric hindrance is involved, due to the large size of the protein molecules. In Additional file 1: Figure S1, we show the interaction interfaces of HSA and Fc with FcRn. As it may be noticed, the two interactors bind at opposite regions of the α1–α2 subunits. As mentioned above, hindrance is not expected to occur since the size of both ligands is quite small. On the other hand, we may postulate that large sized ligands such as exameric fibrinogen may interfere with the binding of both HSA and Fc, either competitively or non-competitively.

Another important aspect is the pH dependency of the binding. Indeed, FcRn binds IgG and albumin at acidic pH, which can be found in early and late endosomes, in the proximal tract of intestine during neonatal life and, eventually, in the extracellular space of inflamed tissues [34]. It is well documented by literature that FcRn is expressed in dendritic cells, where it directs immune complexes to lysosomes for facilitation of antigen presentation, in monocytes/macrophages, in polymorphonuclear leukocytes and also in B lymphocytes [35, 36]. Here we presented clear evidence for the expression of FcRn in T cells as well, where the protein is present in its glycosylated form. A single N-glycosylation site (N102 residue within NTS motif) is present in human FcRn, where the addition of a glycan moiety increases the molecular mass by 1.5–3 kDa [29], which is in keeping with the two discrete protein forms that we detected in promyelocytic and SH-SY5Y cell lines. The biological significance of the addition of glycan moieties to human FcRn remains unclear. *N*-Glycans have been suggested to be apical targeting signals in other proteins [37] and it has been recently proposed that this modification may play a role in mediating apical membrane distribution of FcRn and enhancing either stabilization of FcRn on the cell surface or movement of FcRn to the cell surface [29].

In this context, within sites of inflammation fibrinogen could bind FcRn on the surface of T cells, be internalized, protected from degradation and eventually recycled extracellularly.

## Conclusions

Collectively, the biological and functional implications of our findings are important. Indeed, three of the most abundant proteins (i.e., HSA, IgG and fibrinogen) regulating key processes in the homeostasis of the cell and of the biological fluids, as well as of the immunity of the individual, can be protected by drastic degradation using the FcRn-mediated mechanism. This allows these proteins to increase their half-life and thus prevent an excessive biosynthesis of already highly expressed molecules. It is important to underline that a major cellular component in the blood involved in the internalization and protection from degradation of fibrinogen is the T cell compartment. T cells are involved in crucial functions of the immune response. In particular the CD4+ T cells, which represent up to 40% of the total circulating mononuclear cells, are involved in the initial phases of the adaptive immune response against foreign aggressors by recognizing antigens and subsequently triggering the effector phases of the immune response [38–40]. As such, T cells are among the first cells to infiltrate inflamed tissues to patrol the presence of foreign aggressors. As it has been postulated for granulocytes, during inflammation the requirement of high concentration of fibrinogen may be key in the process of recruitment and extravasation of the inflammatory cells [3, 41]. The fact that lymphocytes, and particularly T-cells, will eventually colonize these tissues to contribute to the elimination of the insult makes them ideal candidates to maintain a high concentration of fibrinogen in situ without the need of continuous support of new protein synthesis by the liver. Future investigation on this aspect is certainly warranted.

## Additional file

**Additional file 1: Figure S1.** 3D model of binding of HSA and Fc with FcRn.

## Abbreviations

BLAST: basic local alignment search tool
CHAPS: 3-[(3-cholamidopropyl)dimethylammonio]-1-propanesulfonate
Ct: cycle threshold
DMEM: Dulbecco’s modified Eagle’s medium
EDTA: ethylene-diamine-tetraacetic acid
FACS: fluorescence activated cell sorting
FBS: fetal bovine serum
FCGRT: Fc fragment of IgG receptor and transporter
FcRn: neonatal Fc receptor
FITC: fluorescein isothiocyanate
HSA: human serum albumin
IgG: immunoglobulin G-type
IL-6: interleukin 6
MACS: magnetic-activated cell sorting
PBMCs: peripheral blood mononuclear cells
PBS: phosphate-buffered saline
PVDF: polyvinylidenedifluoride
qRT-PCR: quantitative reverse-transcription polymerase chain reaction
RPMI: Roswell Park Memorial Institute (culture medium)
SDS-PAGE: sodium-dodecyl-sulphate polyacrylamide gel electrophoresis
siRNA: short interfering RNA
