# Supplementary material for: Neonatal Fc receptor is involved in the protection of fibrinogen after its intake in peripheral blood mononuclear cells
Source: J Transl Med. 2018 Mar 14;16:64. doi: 10.1186/s12967-018-1446-2 (PMC5853075; doi:10.1186/s12967-018-1446-2)
Supplement: Supplementary file 1 — Additional file 1: Figure S1. 3D model of binding of HSA and Fc with FcRn. [file 12967_2018_1446_MOESM1_ESM.docx]

**ADDITIONAL FILE 1**


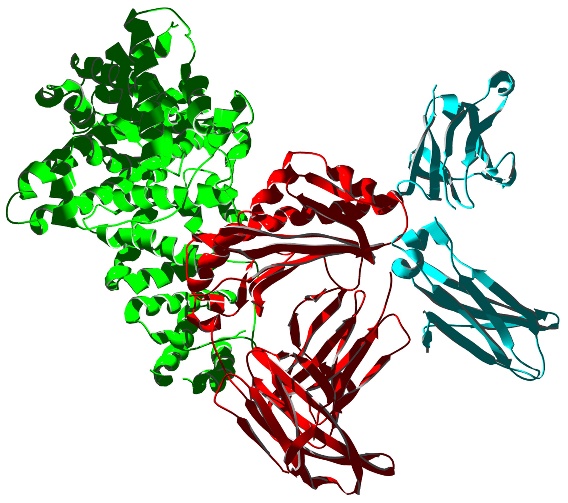


**Additional file 1: Figure S1. 3D model of binding of HSA and Fc with FcRn.** The interaction of FcRn (red) with its two ligands HSA (green) and Fc (cyan) is shown. The model has been obtained by overlapping FcRn coordinates from the structures of a high affinity HSA variant bound to human FcRn (PDB code 4K71) [1] and of the complex of rat FcRn with Fc (PDB code 1FRT) [2]. The structural fit has been computed using SwissPDBViewer [3].

**ADDITIONAL REFERENCES**

1. Schmidt MM, Townson SA, Andreucci AJ, King BM, Schirmer EB, Murillo AJ, et al. Crystal structure of an HSA/FcRn complex reveals recycling by competitive mimicry of HSA ligands at a pH-dependent hydrophobic interface. Struct. Lond. Engl. 1993. 2013;21:1966–78.

2. Burmeister WP, Huber AH, Bjorkman PJ. Crystal structure of the complex of rat neonatal Fc receptor with Fc. Nature. 1994;372:379–83.

3. Guex N, Peitsch MC. SWISS-MODEL and the Swiss-PdbViewer: an environment for comparative protein modeling. Electrophoresis. 1997;18:2714–23.
